# Copper and Nickel Microsensors Produced by Selective Laser Reductive Sintering for Non-Enzymatic Glucose Detection

**DOI:** 10.3390/ma14102493

**Published:** 2021-05-12

**Authors:** Ilya I. Tumkin, Evgeniia M. Khairullina, Maxim S. Panov, Kyohei Yoshidomi, Mizue Mizoshiri

**Affiliations:** 1 Institute of Chemistry, Saint Petersburg State University, 7/9 Universitetskaya Nab., 199034 St. Petersburg, Russia; e.khayrullina@spbu.ru (E.M.K.); m.s.panov@spbu.ru (M.S.P.); 2 Department of Mechanical Engineering, Nagaoka University of Technology, Nagaoka 9402142, Japan; s193097@stn.nagaokaut.ac.jp (K.Y.); mizoshiri@mech.nagaokaut.ac.jp (M.M.)

**Keywords:** selective laser sintering, femtosecond laser, copper, nickel, microsensors, non-enzymatic sensing, glucose

## Abstract

In this work, the method of selective laser reductive sintering was used to fabricate the sensor-active copper and nickel microstructures on the surface of glass-ceramics suitable for non-enzymatic detection of glucose. The calculated sensitivities for these microsensors are 1110 and 2080 μA mM^−1^·cm^−2^ for copper and nickel, respectively. Linear regime of enzymeless glucose sensing is provided between 0.003 and 3 mM for copper and between 0.01 and 3 mM for nickel. Limits of glucose detection for these manufactured micropatterns are equal to 0.91 and 2.1 µM for copper and nickel, respectively. In addition, the fabricated materials demonstrate rather good selectivity, long-term stability and reproducibility.

## 1. Introduction

At present, significant progress in the treatment of many human diseases including diabetes and cancer has been made. Nevertheless, the problem of timely diagnostics of a disease at the initial stages [1,2] and further adequate treatment have become more important. In this regard, particular attention attracts selective identification of various bioanalytes (chemical biomarkers) in human biological fluids and tissues; biosensors are the most effective devices applied for this purpose. Among the many different types of biosensors, electrochemical biosensors are the most distinguished exhibiting low cost, rapid analysis and simple sample preparation [3,4,5]. These devices are useful in sensing of such analytes as folic acid [6], DNA [7], D- and L-amino acids [8], uric acid [9], choline [10], many cancer biomarkers, e.g., cancer-embryonic antigen [11], glucose [12], hydrogen peroxide [13] and others. A large number of works deal with design and development of electrochemical and optical sensors, in which electrocatalytic activity is revealed by the enzyme-free compounds and materials. Indeed, the enzyme-free sensors allows for avoiding the problems in biosensorics caused by the utilization of enzymes (instability in aggressive environments, high cost and the need to immobilize enzymes on some carriers) [14]. The variety of materials can be applied for non-enzymatic sensing [15,16,17,18,19,20,21,22,23]. In this paper, we discuss sensors based on metals and their alloys and/or composites that have deserved a great interest in this scientific field.

It is necessary to mention the methods that are typically used for fabrication of such materials. In recent, 3D printing technologies have become a popular tool for solving a number of scientific problems [24]. Selective laser sintering (SLS) and selective laser manufacturing (SLM) for 3D microstructures are very promising representatives of such modern techniques [25]. In general, SLS is an additive manufacturing method that can be implemented for formation of 3D structures of a given size and shape by heating powders of various materials (plastic, glass, ceramics, metals) with the focused laser beam up to temperature, at which the powder-like particles fuse together providing the porosity control regime. In contrast to SLS, in SLM a powder is heated before the melting point that allows for decreasing the porosity and obtaining a homogeneous system. Therefore, as a rule, SLM is commonly used for fabrication of metallic materials. In this case, this method is recognized as direct laser metal sintering (DLMS). SLM is already actively applied in production of the electrochemical sensors based on metals and their alloys suitable for detection of phenols [26] or explosives and nerve agents [27]. Other noticeable techniques that can be utilized for the aforementioned purposes are: roll-to-roll printing (R2R) [28], inkjet printing [29], screen printing [30], chemical vapor deposition (CVD) [31], laser-induced metal deposition (LCLD) [32], femtosecond laser reductive sintering of metal oxide nanoparticles [25,33,34,35], etc. At some level all these methods demonstrate different drawbacks mostly related to cost, maintenance, complexity, efficiency and others. In turn, selective laser sintering, despite its own disadvantages, provides rather good reproducibility, rapidity and efficiency in fabrication of metallic structures reliable for non-enzymatic sensing. Femtosecond laser reductive sintering is a promising candidate for fabricating the metal patterns because the thick metal patterns can be formed in air by simulatenously metalizing and sintering of metal oxide nanoparticles. In comparison with LCLD, thicker metal patterns are formed by femtosecond laser pulse-induced thermochemical reaction because the intense laser pulses achieve the local heating of the raw metal oxide nanoparticles.

Thus, this work is devoted to the conditions optimization for fabrication of copper and nickel micropatterns using SLS method. We expect that the proposed approach can be applied as a fast, efficient and cheap way to create sensor platforms for enzyme-less detection of low concentrations of various bioanalytes. As a result, we fabricated the glucose microsensors based on such metals as copper (Cu) and nickel (Ni), which are widely used due to their low cost and high electrical conductivity.

## 2. Materials and Methods

The solution with CuO nanoparticles (NPs) (<50 nm particle size, Sigma Aldrich, St. Louis, MS, USA), polyvinylpyrrolidone (PVP, Mw 10000, Sigma Aldrich, St. Louis, MS, USA) and ethylene glycol (EG, 99.8%, Sigma Aldrich, St. Louis, MS, USA). First, PVP were mixed with EG. Subsequently, CuO NPs were dispersed into the mixed solution. The resulting CuO NP-based solution was cover by spin-coater on glass-ceramics substrates (5 s—500 rpm, 30 s—7000 rpm). A similar approach was applied to obtain films based on nickel oxide (NiO) nanoparticles. NiO NP (<50 nm particle size, Sigma Aldrich) were used as a source of Ni. It has been demonstrated that NiO NPs were reduced to Ni by femtosecond laser reduction of NiO NP solutions consisting of NiO NPs, PVP and EG 23. 

All metal structures were produced on glass-ceramic. Glass-ceramic material consists of silicon dioxide (60.5%), aluminum oxide (13.5%), calcium oxide (8.5%), magnesium oxide (7.5%) and titanium dioxide (10.0%).

Surface preparation was carried out using ultrasonic cleaning in acetone, ethanol and water (sequentially), then the dried substrates were treated by irradiating ozone for 1 min to improve the wetting property of the prepared colloidal solution with polarity for each substrate using the FLAT EXCIMER EX-mini (Hamamatsu, Japan), after which the solution was deposited using a spin-coater (MS-B100, MIKASA CO. LTD, Tokyo, Japan).

A femtosecond fiber laser (TOPTICA Photonics AG, Munich, Germany), pulse duration: 120 fs, wavelength: 780 nm, repetition rate: 80 MHz) was used for direct patterning of Cu and Ni microstructures. Femtosecond laser pulses were focused with an objective lens with a numerical aperture of 0.45. First, a solution containing nanoparticles of CuO or NiO was placed on glass ceramics using spin-coating technique. Then, the micropatterns were directly produced by the focused femtosecond laser pulses. The raster pitch of the micropatterns was decided to be 5 µm and 10 µm. 

The crystal structures of the patterns formed by raster scanning of the focused femtosecond laser pulses were examined by an X-ray diffraction (XRD) analysis (Rigaku RINT, MiniFlex) (Rigaku Corporation, Tokyo, Japan) using Cu Kα radiation. 

The topology of Cu and Ni electrodes were investigated using scanning electron microscopy (Zeiss Supra 40 VP, Oberkochen, Germany). The EDX-system was coupled with a scanning electron microscope equipped with X-ray attachment (Oxford Instruments INCA X-act) (Oxford Instruments, Abingdon, UK) was conducted to quantitatively investigate the major chemical composition ratio in the Cu and Ni electrodes.

The electrocatalytic activity of the fabricated Cu and Ni-based materials towards glucose was studied using voltammetric methods (potentiostat, Elins P30I) (Electrochemical Instruments Ltd., Chernogolovka, Russia). In order to increase the adhesion and lifetime of the synthesized materials, we drop-casted 10 µL of Nafion suspension (0.05 wt%) on the Cu- and Ni-based electrodes. All measurements were carried out at room temperature in a typical three-electrode system using a Pt wire as a counter electrode, a Ag/AgCl reference electrode, and pre-air-dried Cu and Ni-based microelectrodes as working electrodes. The solutions of D-glucose of different concentrations were added to background solution (0.1 M sodium hydroxide) with simultaneous stirring.

## 3. Results

**Figure 1:**
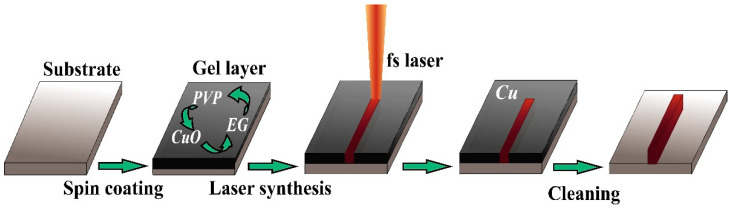
Schematic process of fabrication of metal micropatterns using SLS. A solution containing copper or nickel nanoparticles deposited on a substrate is irradiated with a femtosecond fiber laser (120 fs, Toptica, FemtoFiber pro NIR (780 nm)). The unreacted solution is removed by washing with a solvent.

The optimized compositions for deposition of Cu and Ni micropatterns on glass-ceramic surfaces are presented in Table 1. The regimes of the composition optimization for deposition were previously published for Cu patterns on the glass surface [34]. In the current work, we proposed and optimized a technique for Cu and Ni manufacturing on the surface of glass-ceramics. The main concept of the SLS experiment used in the current study is shown in Figure 1. 

**Figure 2:**
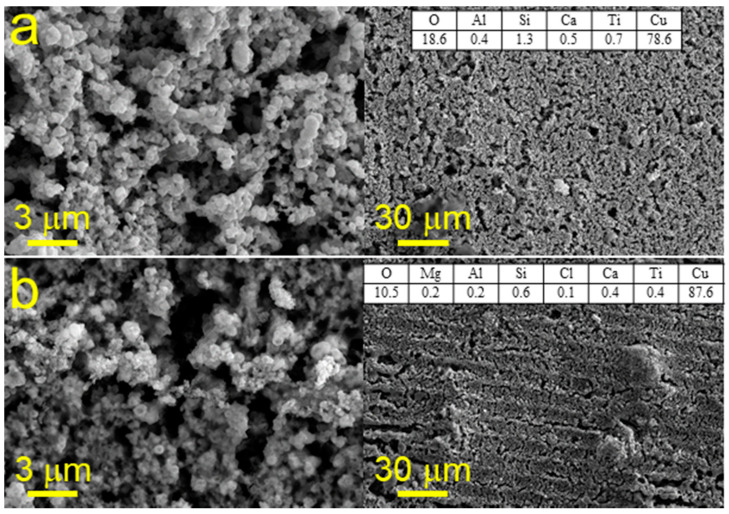
SEM images and results of EDX analysis (right top corner in each panel) of Cu patterns fabricated on glass-ceramics using SLS at the laser fluence of 0.0192 J/cm^2^ along with the scanning speed of 5 (**a**) and 10 (**b**) mm per second.

**Figure 3:**
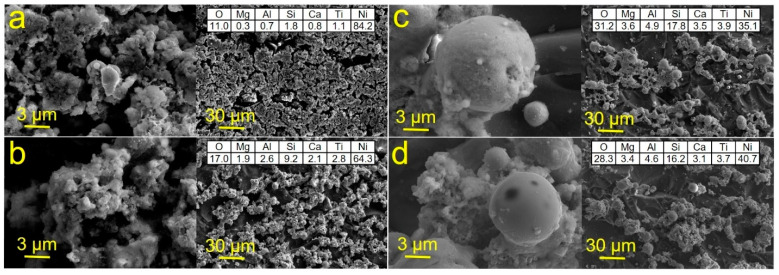
SEM images and results of EDX analysis (right top corner in each panel) of Ni patterns fabricated on glass-ceramics using SLS at the following laser fluences (J/cm^2^) and scanning speeds (mm s^−1^): (**a**) 0.0192 and 5; (**b**) 0.0154 and 5; (**c**) 0.0192 and 10; (**d**) 0.0154 and 10.

In our previous works, it was shown that the main parameters that significantly affect the composition and topology of materials fabricated by the reductive SLS are laser fluence, scanning speed and pitch size. Therefore, by varying these parameters, it is possible to create materials with different functional properties. In this case, we have shown the possibility to produce the working electrodes for enzyme-free electrochemical detection of glucose. For this purpose, we optimized the conditions for synthesis of the conductive coatings on the surface of a dielectric with high adhesion and a developed surface. For fabrication of Cu electrodes on glass, we used the laser fluence varied from 0.0096 to 0.0230 J/cm^2^, whereas the scanning speed was 1–10 mm per second and the distance between the deposited lines was varied between 5 and 10 µm. In turn, the analysis of SEM images of Cu patterns on glass-ceramics has shown that the most optimal conditions for producing homogeneous materials are the following: laser fluence of 0.0192 J/cm^2^, scanning speed of 5 mm per second and distance between lines of 5 µm (Figure 2a). Thus, we were able to produce the metallic film consisting of the 50–300 nm Cu particles. It is important that the most homogeneous film on the surface of glass-ceramics can be deposited at a laser fluence of 0.0192 J/cm^2^ (Figure 2); presumably, due to the more resistant to temperature nature of glass-ceramics in opposite to glass, for which the same value was equal to 0.0154 J/cm^2^. Analysis of the electronic microphotographs of Ni patterns on the surface of glass-ceramics showed that the most homogeneous layer of metal could be obtained at laser fluence of 0.0192 J/cm^2^ (Figure 3). In this regard, we have conducted the SLS experiments at laser fluence varied between 0.0096 and 0.0230 J/cm^2^. At 0.0192 J/cm^2^, Ni is deposited as a thin film consisting of particles with size of 100–500 nm. EDX analysis confirms that the main component of the patterns is Ni.

Summarizing the results of SEM and EDX studies, we found the most optimal conditions for Cu and Ni sintering (Table 2). 

**Figure 4:**
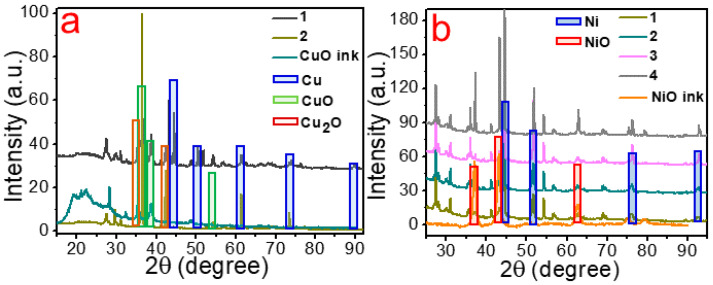
XRD patterns of (**a**) Cu (samples 1, 2 and initial mixture (CuO ink) are shown in the legend) on glass-ceramics and (**b**) Ni (samples 1–4 and initial mixture (NiO ink) are shown in the legend) on glass-ceramics sintered at the following laser fluence (J/cm^2^), scanning speed (mm s^−1^) and pitch size (μm): 0.0192, 5, 5 for 1; 0.0154, 5, 5 for 2; 0.0192, 10, 5 for 3; 0.0154, 10, 5 for 4.

Furthermore, we carried out the phase analysis of Cu and Ni materials obtained using the conditions mentioned before (Figure 4). XRD shows that the synthesized deposits contain metallic Cu and Ni together with a small amount of their oxides. Besides, we also observe peaks associated with the material of a substrate (glass-ceramics). Figure 3a illustrates XRD diffractograms of Cu patterns. As is shown here, for fabrication of patterns enriched with Cu, we need to apply the following conditions: laser fluence of 0.0192 J/cm^2^, scanning speed of 5 mm per second and distance between the lines of 5 µm (Figure 4a, 1). XRD of the sample obtained at a scanning speed of 10 mm per second (Figure 4a, 2) reveals the presence of Cu(I) oxide indicating that the reduction reaction of Cu(II) to its metallic state was incomplete. According to phase studies shown in Figure 4b, we can conclude that the most optimal conditions for fabrication of Ni-rich homogeneous patterns are the following: laser fluence of 0.0192 J/cm^2^ and scanning speed of 5 mm per second.

It should be pointed out here that increasing the pitch size and the scanning speed (up to 10 mm per second) during laser irradiation leads to formation of non-uniform deposits with numerous defects containing metal oxides and, as a result, having a poor electrical conductivity. This could be related to the lack of time and laser fluence to complete metal reduction reaction (for both Cu and Ni). On the other hand, a too-low scanning speed (less than 1 mm per second) contributes to formation of smoother films due to a more complete fusion of the original NPs, resulting in deformation of a deposit and decrease of the surface area and the number of available active electrocatalytic centers.

**Figure 5:**
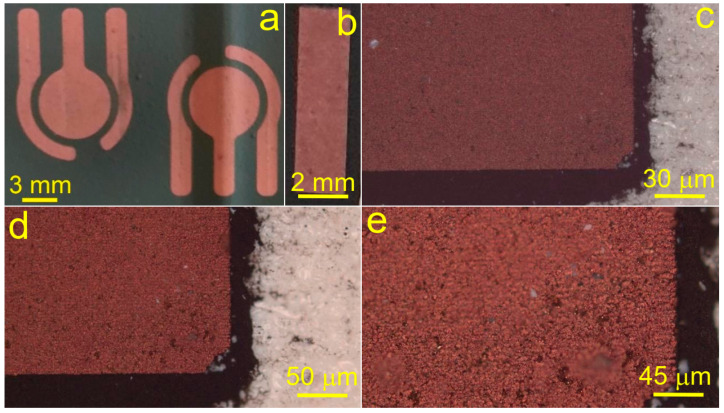
Photographs of copper micropatterns of different geometry fabricated on the surface of glass-ceramics using SLS. (**a**) Three-electrode geometry suitable for design of a sensor platform based on copper; (**b**–**e**) Pictures of copper rectangular microstructures of different scale. Black background corresponds to the area unexposed to the laser irradiation.

Thus, using SLS, it is possible to fabricate the electrodes of various shapes and geometries relying on the optimized parameters. Figure 5 illustrates different geometries of copper microelectrodes obtained on the surface of glass-ceramics. Potentially, these geometries can be used to create a sensor platform based on the three-electrode electrochemical cell (Figure 5a).

**Figure 6:**
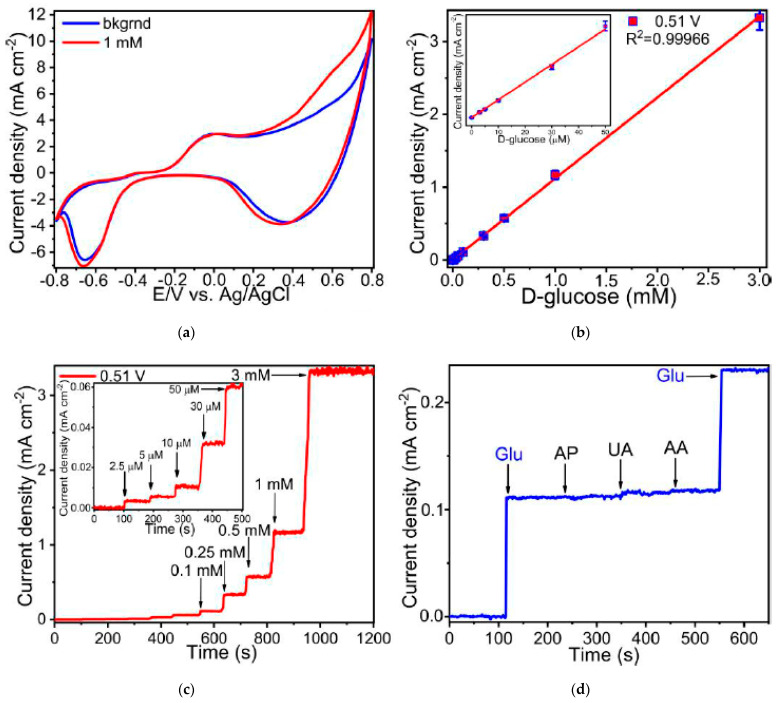
(**a**) Cyclic voltammograms of Cu patterns (electrode) measured in the solution of 0.1 M NaOH with addition of 1 mM D-glucose; (**b**) Amperogram of Cu electrode recorded at potential of 0.51 V in 0.1 M NaOH solution containing of D-glucose of various concentration; (**c**) Linear range of D-glucose concentrations for enzyme-less sensing using the fabricated Cu electrode; (**d**) Selectivity of Cu electrode towards 100 μM D-glucose (Glu) detection in the presence of 20 μM 4-acetamidophenol (AP), 20 μM uric acid (UA) and 20 μM ascorbic acid (AA) observed in the background solution of 0.1 M NaOH. In these experiments, we used Cu electrodes sintered on glass-ceramics.

**Figure 7:**
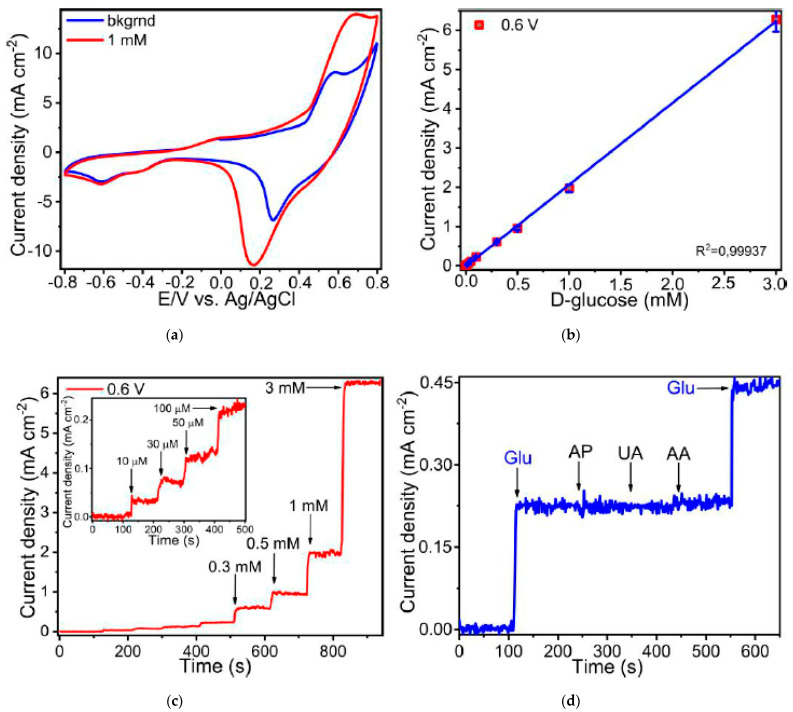
(**a**) Cyclic voltammograms of Ni micropatterns (electrode) measured in the solution of 0.1 M NaOH with addition of 1 mM D-glucose; (**b**) Amperogram of Ni electrode recorded at potential of 0.6 V in 0.1 M NaOH solution containing of D-glucose of various concentration; (**c**) Linear range of D-glucose concentrations for enzyme-less sensing using the fabricated Ni electrode; (**d**) Selectivity of Ni electrode towards 100 μM D-glucose (Glu) detection in the presence of 20 μM 4-acetamidophenol (AP), 20 μM uric acid (UA) and 20 μM ascorbic acid (AA) observed in the background solution of 0.1 M NaOH. In these experiments, we used Ni electrodes sintered on glass-ceramics.

The electrocatalytic activity of the synthesized Cu and Ni micropatterns towards enzyme-free glucose sensing was tested using voltammetric methods. Figure 6a and Figure 7a demonstrate cyclic voltammograms (CVs) of these materials measured in the background solution containing 1 mM D-glucose. The shape of CVs for Cu sintered on glass-ceramics have very broad range of potentials between 0.35 and 0.65 V corresponding to anodic glucose oxidation (Figure 6a). In turn, electrooxidation of glucose on Ni microelectrode takes place within the region of potentials of 0.45–0.7 V, which shifts toward larger potentials with an increase of the glucose concentration (Figure 7a).

All electrochemical characteristics such as sensitivity, limit of detection (LOD), linear range of glucose detection and selectivity were obtained using amperometry. Figure 6b and Figure 7b illustrate the amperometric response to the consecutive additions of D-glucose to 0.1 M NaOH at potentials of 0.51 V for Cu and 0.6 V for Ni. Then, we obtained the linear dependence of the analytical signal (Faraday current) vs. D-glucose concentration for each material (Figure 6c and Figure 7c). According to this data, linear regime of enzymeless glucose detection is provided between 0.003 and 3 mM for Cu, whereas for Ni linear range lies between 0.01 and 3 mM. The sensitivity of the electrodes were estimated by calculating the slopes of a linear curves shown in Figure 6c and Figure 7c. As a result, the calculated sensitivities for Cu and Ni are 1110 and 2080 μA mM^−1^·cm^−2^, respectively. In addition, limits of glucose detection for all manufactured materials were calculated as LOD = 3 S/b, where S is the standard deviation from linearity and b is the slope of the calibration curve indicated in Figure 6c and Figure 7c, and are equal to 0.91 and 2.1 µM for Cu and Ni, respectively. The measurement error does not exceed 7%. In addition, the error in the calculated sensitivities is also very small (R^2^ is close to unity and is 0.9996 and 0.9994 for copper and nickel samples, respectively). Rather high sensitivity values can be explained by the close contact between the electrocatalytically active material and the current collector, since they are a single structure in contrast to the electrodes deposited on a conductive substrate by dropcasting or similar methods. This leads to an increase in electric conductivity and, as a result, sensitivity. The values of the electrochemical parameters observed for Cu and Ni micropatterns in this work in comparison with characteristics of the analogical non-enzymatic sensors are shown in Table 3.

The selectivity of the fabricated materials with respect to glucose sensing was investigated in the presence of such interfering compounds as 4-acetamidophenol (AP), ascorbic acid (AA) and uric acid (UA) that usually coexist with glucose in the human blood (Figure 6d and Figure 7d). Thus, Cu and Ni micropatterns have a good selectivity for glucose sensing exhibiting much more significant analytical response towards D-glucose opposite to other analytes. 

We also studied the long-term stability and reproducibility of the fabricated microelectrodes. Rather good stability was confirmed by testing five samples of each material for 10 days. We observed that during this period all samples maintained ~92–95% their initial electrocatalytic activity with respect to non-enzymatic glucose sensing. On the other hand, the great reproducibility was supported by low values of the relative standard deviation (~5–8% for all samples) of the analytical response to 1 mM D-glucose.

## 4. Conclusions

In this work, we found the optimal parameters for manufacturing Cu and Ni micropatterns on the surface of glass-ceramics using reductive selective laser sintering (SLS). In order to increase the adhesion and lifetime of these materials on the surfaces of the substrate used in the current study, they were treated with Nafion solution. The fabricated Cu and Ni electrode materials can be used for non-enzymatic glucose sensing. It was confirmed by the electrochemical experiments that revealed their high sensitivity (1110 and 2080 μA mM^−1^·cm^−2^), low limit of detection (0.91 and 2.1 µM), broad linear range (0.003–3 mM and 0.01–3 mM), as well as good selectivity and long-run stability. Thus, it is possible to conclude that high speed reductive SLS allowing for obtaining electrodes of various geometries is a quite promising technique for the design and fabrication of reliable materials for enzyme-free sensing purposes that can compete with existing technologies used for the production of microelectronic devices and sensors.

## Figures and Tables

**Table 1:** The optimized compositions for deposition of Cu and Ni micropatterns on glass-ceramic.

Material of Electrode	CuO or NiO, g	PVP, g	EG, g
Cu	3	0.65	1.35
Ni	1.5	0.65	1.35

**Table 2:** The optimized SLS experimental conditions used for synthesis of Cu and Ni on glass-ceramics surfaces.

Material of Electrode	Laser Fluence, J/cm^2^	Scanning Speed, mm/s	Pitch Size, µm
Cu (Glass) [35]	0.0154	5	5
Cu (Sitall)	0.0192	5	5
Ni (Sitall)	0.0192	5	5

**Table 3:** Different electrode materials for non-enzymatic glucose sensing in comparison with those fabricated in this work.

Electrode Material	Sensitivity (μA mM^−1^ cm^−2^)	Linear Range (mM)	Limit of Detection (μM)	Ref.
Cu on glass-ceramics	1110 ± 6,45	0.003−3	0.91	This work
Ni on glass-ceramics	2080 ± 18,53	0.01−3	2.1	This work
Cu MPs	2432	0−4.711	0.19	[36]
Cu coating	2149.1	0.001−4.6	0.03	[37]
carbon electrode/nanoporous Cu	33.75	0.0006−3.369	2.6	[38]
Cu NPs	412	0−0.7	2.76	[39]
Ni NPs on carbon nanotubes	1438	0.001−1	0.5	[40]
rhizobia-like Ni NPs	50.97	0.001−7	0.18	[41]
Ni NP/chitosan	318.4	0−9	4.1	[42]
3D porous carbon/Ni NPs	207.3	0.015−6.45	4.8	[43]

## Data Availability

The data presented in this study are available on request from the corresponding author.
